# Improving lung cancer screening in real-world settings: a pragmatic approach to provider education, barriers, and solutions

**DOI:** 10.3389/fonc.2026.1750327

**Published:** 2026-03-25

**Authors:** Francesca C. Duncan, Alejandro J. Carrasco, Allie Carter, Marisa Pietrandrea, Edwin J. Jackson, Catherine R. Sears

**Affiliations:** 1 Division of Pulmonary, Critical Care, and Sleep Medicine, Indiana University School of Medicine, Indianapolis, IN, United States; 2 Department of Medicine, Indiana University School of Medicine, Indianapolis, IN, United States; 3 Department of Biostatistics and Health Data Science, Indiana University School of Medicine, Indianapolis, IN, United States; 4 Department of Pediatrics, Indiana University School of Medicine, Indianapolis, IN, United States; 5 Richard Roudebush Veterans Administration Medical Center, Indianapolis, IN, United States

**Keywords:** barriers to lung cancer screening, early detection, implementation science, lung cancer screening, practice-based interventions, primary care, provider education, safety-net hospital

## Abstract

**Introduction:**

Lung cancer is the leading cause of cancer mortality in the United States. While lung cancer screening (LCS) with low-dose computed tomography (LDCT) has been shown to have a 20% relative reduction in mortality, the real-world benefit of screening has not been realized as screening of eligible individuals remains low. Primary care providers (PCPs) play a pivotal role in identifying eligible patients, initiating shared decision-making, and coordinating follow-up care, yet many report limited knowledge and confidence in implementing LCS. Provider education is a critical, scalable intervention to address these barriers and improve screening delivery in primary care. Pilot pragmatic trials are essential for evaluating real-world strategies to increase LCS uptake in primary care settings.

**Methods:**

We utilized a clinical advisory board of LCS experts to co-develop a 30-minute LCS education curriculum tailored for primary care providers (PCPs) at a safety-net hospital in Central Indiana and assessed its’ impact on PCPs’ knowledge of LCS guidelines, comfort with shared decision-making (SDM), communicating LDCT results, and confidence in managing post-screening care.

**Results:**

Following completion of the LCS education curriculum, primary care providers reported increased comfortability discussing LCS and LDCT results with their patients and increased familiarity with LCS eligibility criteria, which was reflected by improved application of screening guidelines to patient scenarios. LCS referrals by primary providers also increased following implementation of the LCS education curriculum.

**Discussion:**

Implementation of the LCS education curriculum led to improved provider comfort in discussing LCS with patients, confidence in managing next steps following LDCT results, increased knowledge of LCS eligibility, and application of screening criteria to patient scenarios, which was associated with an increased number of new referrals of eligible patients to the lung cancer screening program. Future aims can be developed to expand a LCS education curriculum beyond a single primary care clinic and digitize the curriculum into interactive modules, microlearning formats, and mobile-friendly platforms to enhance accessibility for providers.

## 1 Introduction

Lung cancer is the leading cause of cancer-related death in the United States, accounting for an estimated 124,730 deaths and 20% of all cancer-related deaths in 2025 (1). In the United States, the overall 5-year survival rate for lung cancer is 29.7%, underscoring its persistently poor prognosis despite recent improvements (2). Lung cancer’s poor prognosis is largely due to its frequent diagnosis at advanced stages, when curative treatment options are limited (3). However, when detected at an early stage, 5-year survival can exceed 60% (3). Unfortunately, fewer than one-third of lung cancer cases in the United States are diagnosed at an early stage (3). Detecting lung cancer at an early-stage of disease when treatment is curative can substantially improve prognosis and survival (4, 5). This was demonstrated by the National Lung Screening Trial (NLST), the largest randomized lung cancer screening (LCS) controlled trial to date (4). LCS using low-dose computed tomography (LDCT) demonstrated a 20% relative reduction in mortality in high-risk individuals when compared to screening with chest X-rays (4). Unfortunately, despite clear evidence supporting LCS and strong recommendations from organizations such as the United States Preventative Service Task Force (USPSTF) and coverage by the Centers for Medicare and Medicaid Services (CMS) (6, 7), adherence to screening guidelines and implementation of LCS remains low, with national rates as low as 18%, with marked state-state variation (8). This shortcoming in LCS is further compounded by screening disparities among marginalized, low-income, rural, and uninsured patient populations (9, 10).

Various studies have investigated why LCS rates remain low, with more recent research indicating that low utilization of LCS is in part due to primary care providers’ (PCPs) limited knowledge of eligibility criteria, time constraints for shared decision-making (SDM), lack of insurance coverage, skepticism about the effectiveness of screening, and uncertainty regarding follow-up care (11–13). However, PCPs, who often have well-established, trustworthy relationships with their patients, are well-positioned to engage in SDM regarding LCS and help guide informed screening decisions. Various generalized strategies to increase LCS among high-risk individuals and address provider barriers have been proposed, however, implementing these strategies on a large scale can be challenging as it is not yet known which strategies are feasible, acceptable, and effective. Pragmatic trials are crucial for implementing and evaluating approaches to increase LCS in real-world primary care settings. Such studies could determine how to best implement proposed strategies, which aspects are most useful in resource limited settings, and whether such strategies will require tailored efforts for populations experiencing barriers to care, before implementing them in larger populations. To address these challenges, we hypothesized that a two-way learning model could enhance PCP knowledge and comfort with LCS, while also identifying barriers to utilization, and proposed strategies to increase LCS utilization within the primary care setting. Therefore, this study piloted a provider-facing LCS educational curriculum in a primary care setting, with the primary aim of assessing changes in provider knowledge of screening guidelines, comfort with shared decision-making (SDM) conversations, and confidence in managing LDCT results and follow-up. Through this process, we also engaged in two-way learning by eliciting provider-identified barriers and proposed strategies to improve LCS uptake. As a secondary outcome, we evaluated early signals of curriculum effectiveness by comparing LCS referral patterns before and after implementation.

## 2 Methods

### 2.1 Study design and setting

**Figure 1:**
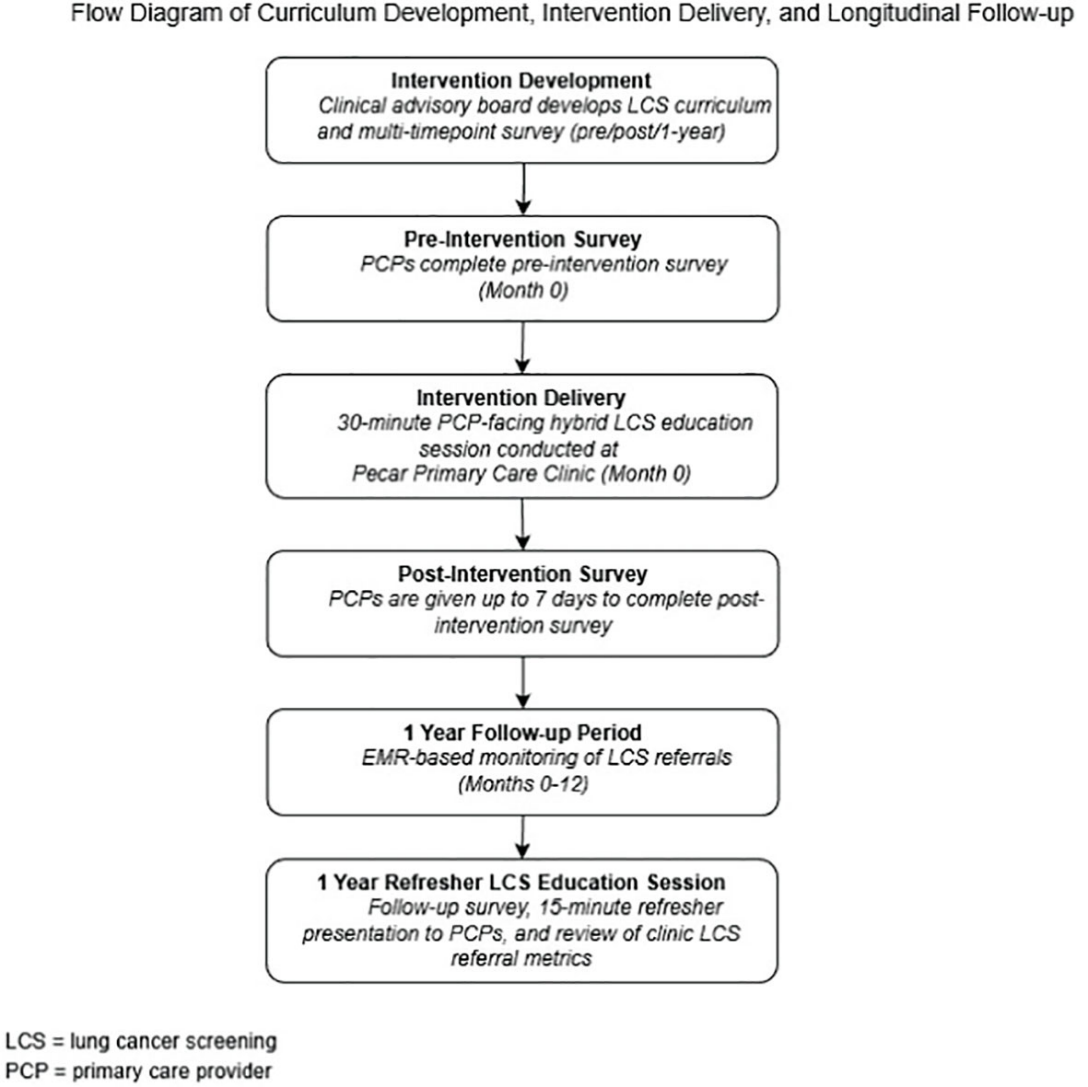
Schematic overview of the provider education intervention, illustrating the timeline of curriculum development, delivery of the training session, and administration of the pre‑, post‑, and 1‑year follow‑up surveys used to assess implementation outcomes.

We conducted a pragmatic pilot study to evaluate the feasibility and preliminary impact of a provider-facing LCS education curriculum in a safety-net primary care setting. Institutional review board (IRB) approval was obtained from Indiana University School of Medicine (18172) prior to initiation of this study. The study was implemented at Eskenazi Health, one of the nation’s largest safety-net community health systems, serving a racially and socioeconomically diverse patient population in Central Indiana. The intervention and data collection occurred between May 2023-May 2024. A study flow chart (**Figure 1**) is included to illustrate the intervention development process, implementation timeline, and follow-up activities.

### 2.2 Curriculum development

The 30-minute education curriculum was co-developed by a 5-member clinical advisory board of LCS experts, including pulmonologists, an implementation scientist, and a local LCS program director. Advisory board members contributed to defining key learning objectives, refining content for accuracy and feasibility in primary care settings, and shaping the final training format through iterative review. Members also reviewed preliminary evaluation results and provided interpretive guidance to ensure findings were clinically meaningful and contextually grounded. Content themes included an overview of lung cancer epidemiology and disparities, evidence for LCS and mortality benefit based on landmark trials, eligibility criteria for screening, SDM communication strategies to introduce LCS to patients, specifics about our local LCS program pipeline, and post-screening care and management. Delivery was tailored to the workflow and constraints of the safety-net primary care clinic. Details on how the curriculum was delivered are described below.

### 2.3 Participants and recruitment

The study was conducted at the Pecar Primary Care Clinic, a satellite clinic of Eskenazi Health, selected for its racially and socioeconomically diverse patient population and inclusion of multiple primary care specialties, specifically internal medicine and internal medicine-pediatrics. All PCPs at the Pecar clinic were invited to participate via email, which included a brief description of the study and a link to the pre-intervention survey. The survey contained an initial question to assess participants’ consent, and participants were informed that they would be asked to complete a pre-intervention, post, and 1-year follow-up survey. Participation was voluntary, and no compensation was provided. Eligible participants included attending physicians, advanced practice providers, physician assistants, resident physicians, medical assistants, nurses, and pharmacists, practicing in adult primary care. Recruitment occurred through email and in-person invitations at the clinic meeting. Provider participation was supported by the Chief Executive Physician at Pecar Primary Care Clinic, a clinic manager, and site coordinator, who served as champions throughout the intervention.

### 2.4 Intervention delivery

The LCS education curriculum consisted of a single 30-minute PowerPoint hybrid in-person and videoconferencing format, delivered during a routine morning provider meeting prior to the start of the clinic day. This was facilitated by the lead author (F.D.) and was the only training session required to fit the time constraints and workflow of the safety-net primary care clinic. Providers were asked to complete the pre-intervention survey via email prior to attending the in-person session and given up to 7 days after the education intervention to complete the post-survey, which was provided via a QR code at the end of the session. As part of the study design, F.D. conducted a brief in-person refresher session (approximately 15 minutes) 1 year after introduction of the curriculum, to reinforce key concepts and share aggregate LCS metrics from the clinic’s 1 year of implementation. This follow-up meeting did not introduce new training content. The 1-year follow-up survey was distributed via email, which included a link to the survey for completion. Of the 43 providers invited to participate in the education curriculum session, 24 attended and completed the pre-intervention survey, 19 completed the post-intervention survey, and 11 completed the 1-year survey representing the intervention’s reach within the clinic.

### 2.5 Data collection, measures, and analysis

This pilot study aimed to assess the feasibility and preliminary impact of a provider-facing LCS education curriculum in a safety-net primary care setting. Feasibility was evaluated based on successful delivery of the curriculum within existing clinic workflows and completion of pre- and post-intervention surveys by participating providers. To assess changes in provider knowledge, confidence, and comfort related to LCS, participants completed structured surveys immediately before, after, and 1-year following the intervention. Survey items included multiple-choice knowledge questions based on current LCS guidelines and Likert-scale assessments for comfort with SDM, communication of LDCT results, and confidence in managing post-screening care. Additional survey items assessed perceived barriers to LCS implementation and strategies to increase uptake, adapted from prior literature and stakeholder input. To explore early indicators of implementation effectiveness, we extracted the number of LCS referrals submitted by providers from the electronic medical record (EMR) during the 3 months before and up to 12 months after curriculum delivery.

Descriptive statistics were used to summarize survey responses, participant characteristics, and referral data as frequencies and percentages. Comparisons were run between pre- *vs*. post-intervention and pre- *vs*. 1 year follow-up timepoints using Fisher’s Exact test. Ordinal variables were compared using Mantel-Haenszel Chi-Square test. Similarly, responses were tested across all three timepoints. Frequencies of provider-selected barriers to LCS implementation and strategies to improve uptake were tabulated to identify common implementation challenges and opportunities. Specific survey items allowed for multiple selections per respondent. These analyses were intended to inform feasibility and preliminary effectiveness of the curriculum to guide future implementation across additional primary care clinics.

## 3 Results

### 3.1 Setting and participant characteristics

Eskenazi Health, located in downtown Indianapolis, Indiana, serves as the state’s oldest and largest safety-net healthcare system. The network provides care across a distributed system of facilities, including the Pecar Primary Care Clinic, a satellite site where this intervention was piloted. The Eskenazi Health Pecar Primary Care Clinic, located in Indianapolis, serves a racially and ethnically diverse population including 53% Hispanic, 34% Black, and 34% White patients across 38,000 annual outpatient visits. Most patients are insured through Medicaid, with self-pay representing the next largest coverage category. Given the disproportionate burden of lung cancer, including higher rates of advanced-stage diagnosis and poorer outcomes among Black and Hispanic individuals, this clinic was strategically selected to ensure the intervention reached populations most affected by the disease and most likely to benefit from efforts to increase screening and early detection. To contextualize the intervention setting, we next describe the demographic characteristics of the primary care providers who participated in the lung cancer screening education curriculum.

As shown in **Table 1**, participants included providers trained in Family Medicine, Internal Medicine, Internal Medicine-Pediatrics, and Obstetrics & Gynecology. This information was missing from 8 individuals. The majority of participants were Attending Physicians, Physician Assistants, and Advanced Practice Nurses, with residents and other roles also included. The proportion of specialties and roles represented in the pre-, post- and 1 year follow-up surveys varied. Most providers reported referring one to three eligible patients per month to the LCS program. Monthly clinic schedules varied, with participants working between one to five full clinic days a month or more than six full days per month. Most indicated that their patients never initiated discussions about LCS.

**Table 1:** Characteristics of primary care providers participating in the lung cancer screening education intervention.

Characteristics of survey participants	Pre-education intervention (n=24)	Post-education intervention (n=19)	1-year follow-up (n=11)	p-value
What specialty do you currently practice in?				0.500
Family Medicine	6 (38%)	10 (63%)	3 (27%)	
Internal Medicine	3 (19%)	2 (13%)	2 (18%)	
Internal Medicine-Pediatrics	6 (38%)	3 (19%)	6 (55%)	
Obstetrics & Gynecology	1 (6.3%)	1 (6.3%)	0 (0%)	
Missing	8	3	0	
What is your role?				0.071
Advanced practice nurse	2 (13%)	3 (19%)	3 (27%)	
Attending Physician	6 (38%)	5 (31%)	4 (36%)	
Resident	0 (0%)	0 (0%)	3 (27%)	
Other	8 (50%)	8 (50%)	1 (9.1%)	
Missing	8	3	0	
On average, how many times per month do you refer eligible patients to the lung cancer screening program?				0.421
Never	5 (29%)	5 (31%)	0 (0%)	
one to three	8 (47%)	9 (56%)	8 (73%)	
four to seven	3 (18%)	2 (13%)	3 (27%)	
>8	1 (5.9%)	0 (0%)	0 (0%)	
Unknown	7	3	0	
On average, how many times per month does a patient ask about lung cancer screening?				0.677
Never	14 (82%)	11 (69%)	8 (73%)	
one to three	3 (18%)	5 (31%)	3 (27%)	
Unknown	7	3	0	
On average, how many clinic days a month do you work?				0.315
< 1 full day	3 (18%)	3 (19%)	0 (0%)	
one to five full days	7 (41%)	8 (50%)	5 (45%)	
> 6 full days	7 (41%)	5 (31%)	6 (55%)	

### 3.2 Primary care provider lung cancer screening knowledge

**Figure 2:**
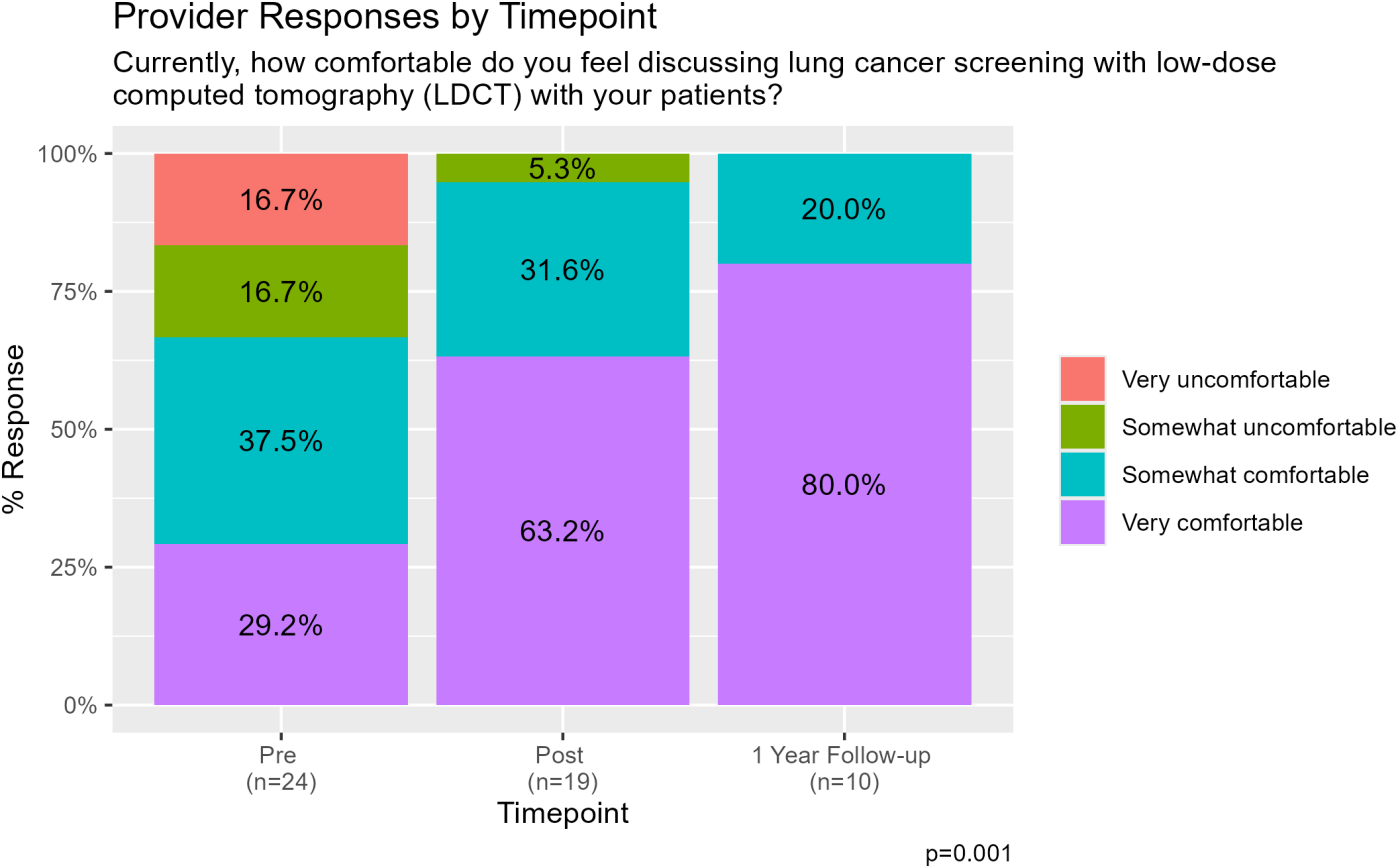
Provider responses to the survey item assessing comfort discussing lung cancer screening with patients, across the pre-, post-, and 1-year follow-up time points.

**Figure 3:**
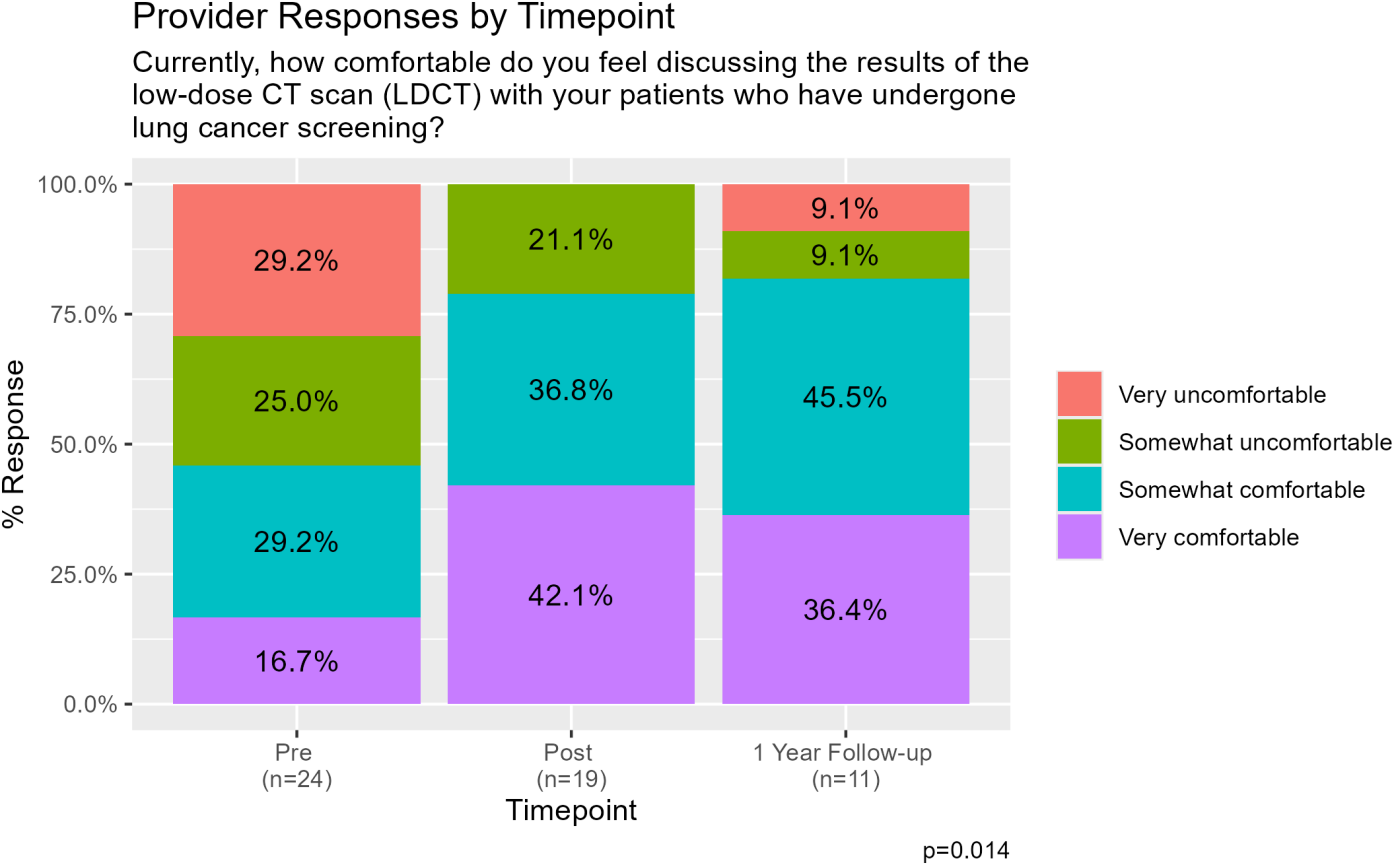
Provider responses to the survey item assessing comfort discussing low-dose CT results with patients who have undergone lung cancer screening, across the pre-, post-, and 1-year follow-up time points.

As shown in **Figure 2**, prior to participation in the LCS education curriculum, 33.4% of practitioners felt “very uncomfortable” or “somewhat uncomfortable” with discussing LCS. Immediately after the education, only 5.3% felt “somewhat uncomfortable” and by 1 year follow-up, no respondents felt uncomfortable discussing LCS. Similarly, only 29.2% of primary care providers reported they felt “very comfortable” discussing LCS with patients prior to the education intervention, as compared to 63.2% reporting feeling “very comfortable” discussing LCS immediately following the education intervention. At 1 year follow-up, 80.0% were “very comfortable” discussing LCS (p=0.001). **Figure 3** shows participant comfort with discussing results of LCS LDCT with their patients. Most (54.2%) felt uncomfortable discussing LDCT results with their patients pre-intervention, with 29.2% very uncomfortable. This improved after the education intervention, with 21.1% feeling “somewhat uncomfortable” and none very uncomfortable on the immediate post-survey. These results persisted at 1 year, with only 9.1% responding they were “very uncomfortable” and 9.1% “somewhat uncomfortable” discussing LDCT results at that time. The percentage of PCPs that felt “very comfortable” and “somewhat comfortable” discussing the results of LDCT with their patients increased from 45.9% of participants prior to the curriculum to 78.9% of participants immediately following the curriculum and 81.9% of participants at 1-year follow-up (p=0.014). Furthermore, confidence in next steps in management following LDCT for LCS increased from 50.0% of PCPs reporting feeling “very confident” and “somewhat confident” to 84.2% immediately following the curriculum and 90.9% at 1-year (**Figure 1**).

**Figure 4:**
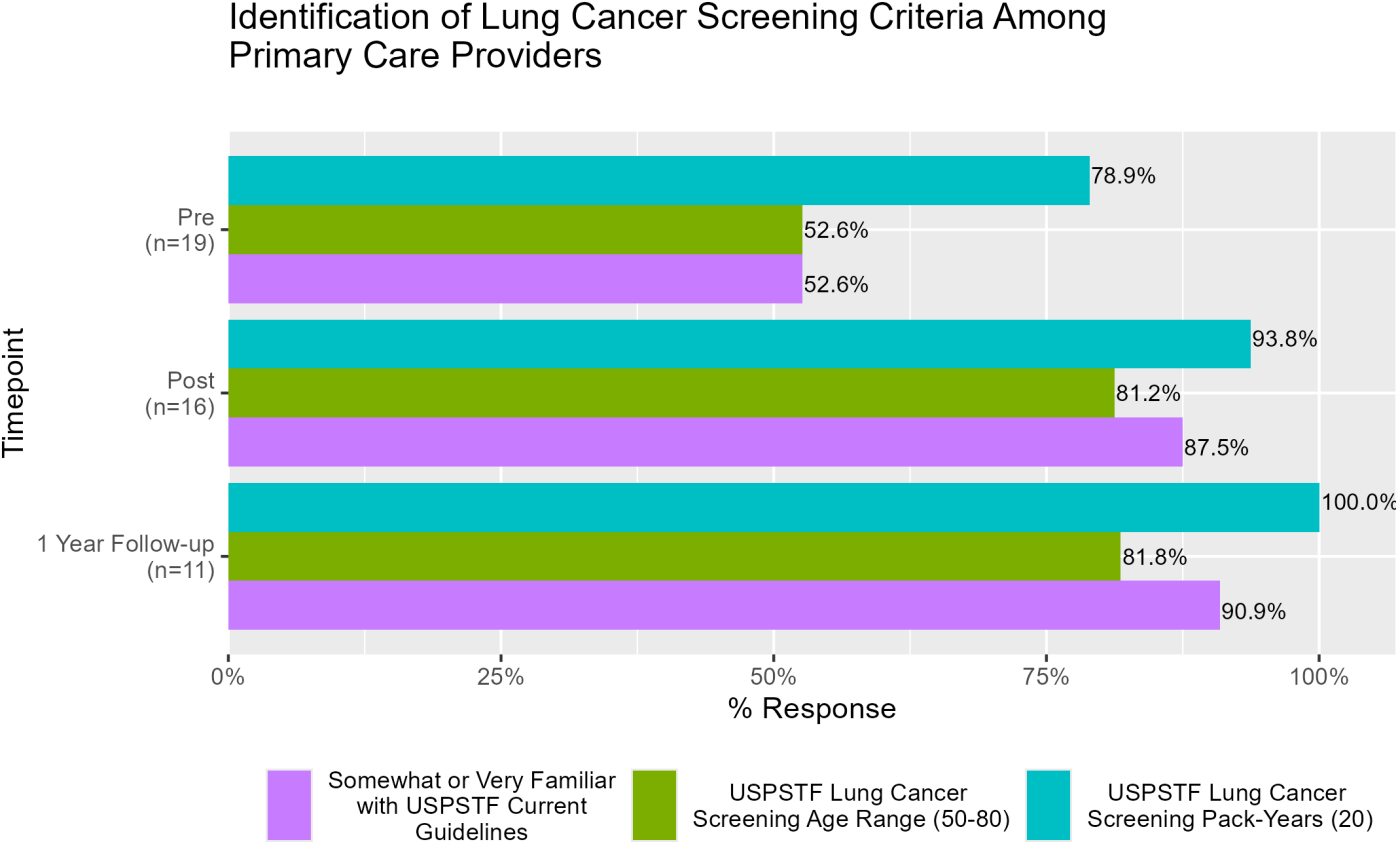
Provider responses to the survey item assessing their ability to correctly identify lung cancer screening eligibility criteria, across the pre-, post-, and 1-year follow-up time points.

Respondents’ knowledge of LCS was tested as well. As shown in **Figure 4**, 52.6% of participants reported familiarity with the United States Preventive Services Taskforce (USPSTF) LCS guidelines prior to the education intervention, which increased to 87.5% and 90.9% immediately post- and at the 1-year follow-up, respectively. There was a 28.6% increase in PCPs’ ability to correctly identify the USPSTF screening age range immediately following the education and remained persistently increased by 81.8% of respondents at 1 year. Furthermore, at 1-year follow-up, 100% of participants were able to correctly identify the pack-years LCS inclusion criteria, which increased from 78.9% pre-intervention.

**Figure 5:**
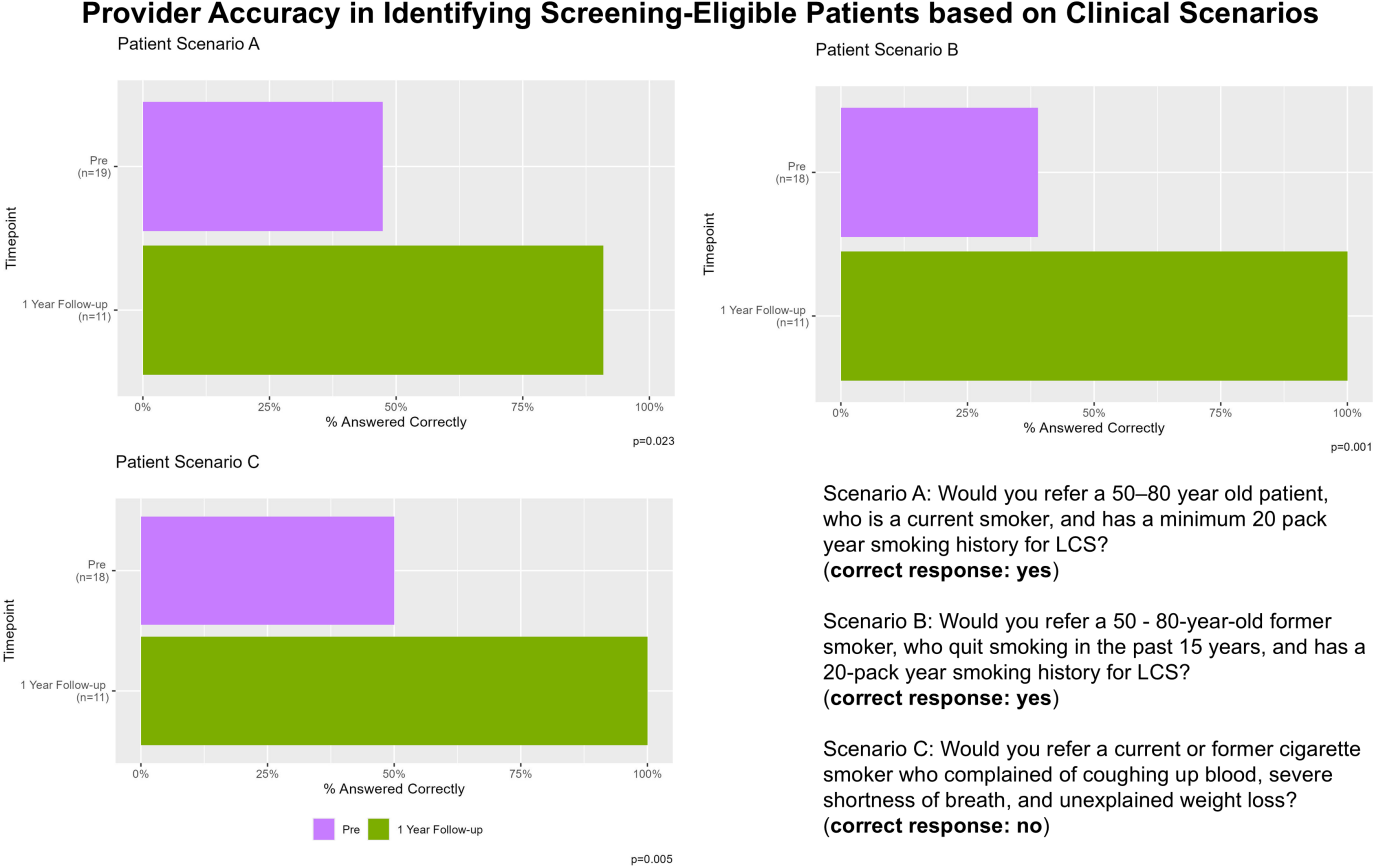
Provider accuracy in identifying lung cancer screening eligible patients based on brief clinical scenarios, across the pre-, post-, and 1-year follow-up time points.

We tested the ability of PCPs to apply LCS criteria to specific patient scenarios. As shown in **Figure 5**, this improvement in knowledge was further supported by an increase in correct responses by PCPs when given three question-based patient scenarios requiring application of the USPSTF LCS guidelines. Scenario A: Would you refer a 50–80 year old patient, who is a current smoker, and has a minimum 20 pack year smoking history for LCS? Scenario B: Would you refer a 50–80 year old former smoker, who quit smoking in the past 15 years, and has a 20-pack year smoking history for LCS? Scenario C: Would you refer a current or former cigarette smoker who complained of coughing up blood, severe shortness of breath, and unexplained weight loss? In patient scenarios B and C, participants demonstrated 100% accuracy, not only in identifying individuals eligible for LCS, but also in recognizing those who were symptomatic and therefore inappropriate candidates for screening, an improvement compared to pre-education intervention (p=0.001, p=0.005, respectively). This distinction is critical to effective shared decision-making and underscores the importance of provider education in avoiding potential harms and ensuring appropriate diagnostic evaluation.

### 3.3 Preliminary effectiveness: impact of LCS education on screening referral patterns

To assess the preliminary effectiveness of the education intervention, we examined changes in lung cancer screening (LCS) referrals from the Pecar Primary Care Clinic over a 12-month period following implementation. In the three months preceding the intervention, a total of 17 LCS referrals were submitted. In contrast, 35 new referrals were made in the first three months post-intervention, 6 in month one, 12 in month two, and 17 in month three, representing a 49% increase compared to the pre-intervention period.

Over the full 12-month post-intervention period, providers submitted 188 new LCS referrals. Of these, 33 were for Hispanic patients and 154 for non-Hispanic patients; 102 referrals were for Black patients and 63 for White patients. These referral patterns suggest that the education intervention may have contributed to increased provider engagement in LCS and expanded access to screening among racially and ethnically diverse populations disproportionately affected by lung cancer burden, suggesting that the intervention may have contributed to more equitable screening outreach.

### 3.4 Provider-identified challenges to LCS uptake and delivery

**Figure 6:**
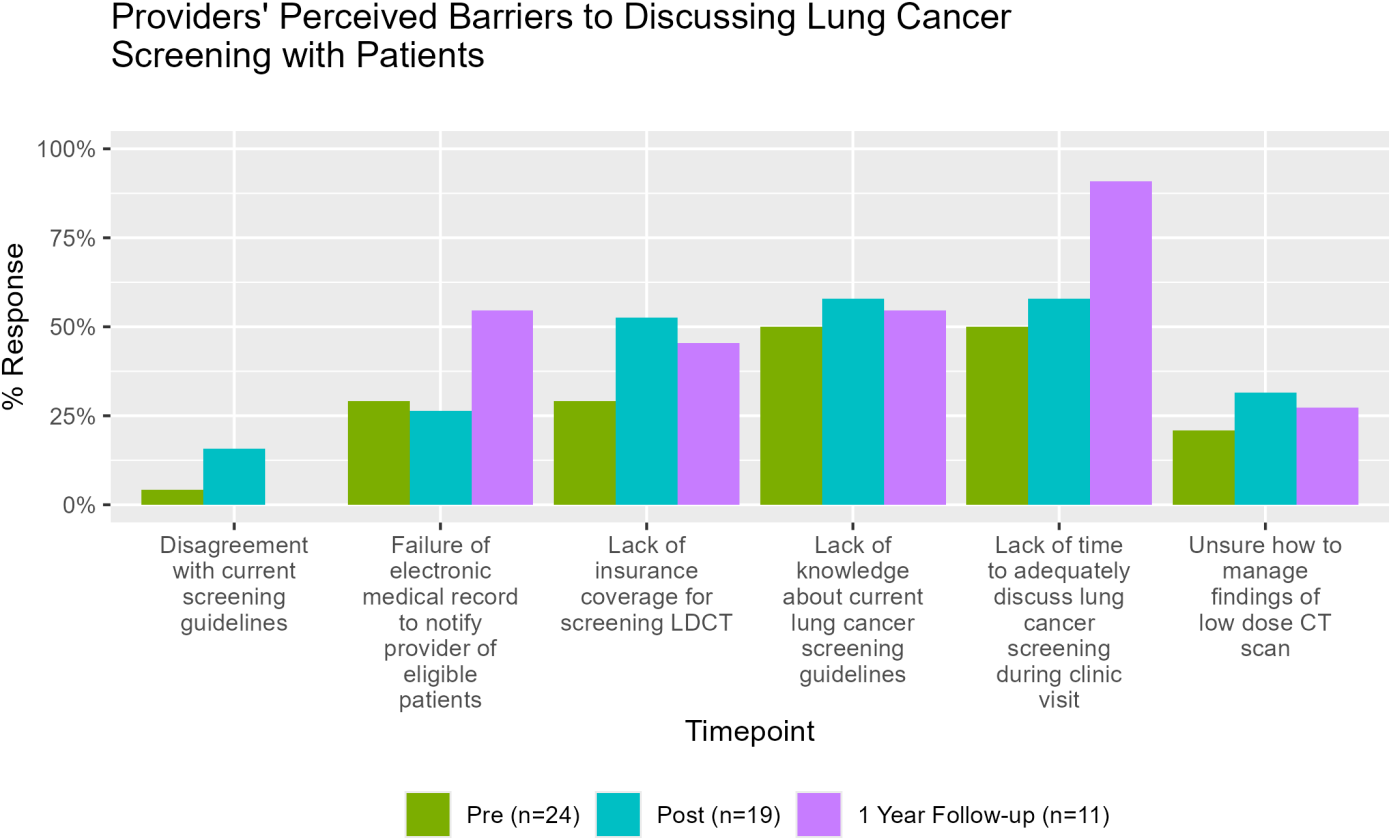
Provider responses to the survey item assessing perceived barriers to discussing lung cancer screening with patients.

When asked to identify barriers to LCS utilization in primary care, PCPs most frequently cited limited knowledge of current LCS guidelines, insufficient time during clinic visits to engage in SDM, and concerns about insurance coverage for low-dose computed tomography (LDCT) LCS (**Figure 6**). At the 1-year follow-up, 87% of participants continued to report lack of time for SDM as a persistent barrier to LCS utilization in the primary care setting, underscoring the need for workflow-integrated solutions to support guideline concordant screening delivery in primary care.

### 3.5 Provider proposed strategies to improve LCS uptake in primary care settings

When PCPs were asked to identify strategies to improve LCS utilization within the primary care setting of a safety-net hospital, providers most frequently endorsed system-level and team-based solutions. These included integrating electronic medical record (EMR) reminders to prompt screening eligibility, developing a standardized algorithm to guide follow-up recommendations for nodule management of LDCT results, assigning a dedicated nurse manager to support patient communication around scheduling, sharing results, and next steps in management following completion of LDCT, and implementing clinic-wide education to ensure all team members are informed about LCS guidelines and workflows (**Figure 2**). These provider-informed strategies reflect key implementation targets to enhance screening delivery in resource-constrained settings.

## 4 Discussion

Despite strong evidence that LCS reduces lung cancer mortality, real-world uptake remains low, particularly among Black and Hispanic populations who are disproportionately affected by advanced-stage disease and worse mortality (4, 9, 10). Prior work by Duncan et al. has shown that Black individuals are significantly more likely to be diagnosed with advanced stage non-small cell lung cancer, have worse mortality, and less likely to receive guideline-concordant treatment (14), reinforcing the need for upstream interventions that promote early detection through LCS and equitable access to care.

This pilot study was designed to address these disparities by implementing a pragmatic, provider-facing education intervention in a safety-net primary care clinic serving a racially and ethnically diverse patient population. The intervention reached populations most affected by lung cancer burden and most likely to benefit from increased screening and early detection. Our findings demonstrate that targeted education can lead to measurable improvements in provider knowledge, confidence, and comfort discussing LCS, and critically, to a marked increase in screening referrals.

Provider knowledge of lung cancer screening guidelines significantly improved following the education intervention and remained high at one-year follow-up. Importantly, providers demonstrated high accuracy in identifying both screening-eligible individuals and those who were symptomatic and therefore inappropriate candidates for screening, a distinction essential to safe and effective shared decision-making. Our findings underscore the value of education not only in promoting uptake, but in ensuring appropriate clinical judgment and minimizing potential harms.

Providers also identified persistent barriers to LCS implementation, including limited time for SDM conversations, lack of familiarity with current guidelines, and concerns about insurance coverage. At one-year follow-up, 87% of participants continued to cite time constraints as a major barrier. These findings align with known implementation challenges and highlight the need for workflow-integrated solutions. Recent reviews highlight persistent barriers to LCS across cognitive, psychological, provider, and system levels, with limited uptake among underserved populations despite proven mortality benefits (15, 16). Our study addresses some of these provider-level gaps by co-developing and pilot-testing a stakeholder-informed educational intervention that offers a scalable, equity-focused strategy to improve LCS knowledge, SDM, and utilization in primary care. Unique to our study is that we also used a two-way learning model where providers were also asked to propose actionable strategies to improve LCS delivery, and responses included EMR-based reminders, standardized follow-up algorithms, dedicated nurse managers for patient communication, and clinic-wide education. These team-based and system-level recommendations reflect key implementation targets and offer a roadmap for scaling interventions in resource-constrained settings.

Our findings are consistent with prior studies demonstrating that provider focused education can improve knowledge, confidence, and delivery of lung cancer screening in primary care settings. One study by Akhtar et al. included the implementation of an education program at two federally qualified health centers which reported increases in provider and medical assistant knowledge as well as higher referral rates following training, underscoring the value of brief, targeted interventions. By contributing evidence from a pragmatic pilot in a safety-net primary care clinic, our study adds to this growing body of work and reinforces the importance of scalable, workflow educational strategies to strengthen LCS implementation.

According to Agurs-Collins et al, multilevel interventions must be thoughtfully designed to meet the needs of populations often excluded from routine screening and preventive care (17). This approach is further supported by the NIMHD Research Framework, which offers a structured lens for examining health determinants across domains (e.g., biological, behavioral, environmental, sociocultural) and levels of influence (18). Our team has operationalized these principles by prioritizing PCP education as a key implementation barrier, recognizing providers’ unique position at the intersection of patient- and system-level determinants (19). The inclusion of a one-year refresher curriculum also provides insight into the potential role of timely reinforcement in sustaining provider engagement, an area not well explored in current literature.

### 4.1 Strengths and limitations

This pilot study offers several strengths. It provides a real-world application of provider-facing LCS education, tailored for PCPs who play a central role in SDM. Longitudinal follow-up surveys allowed for assessment of both immediate and sustained impacts on provider knowledge, confidence, and referral behavior. The increase in referrals and the inclusion of provider-reported barriers and proposed solutions enhance the practical relevance of the findings.

Limitations include the single-site design, which may limit generalizability. The absence of a control group restricts causal inference, and self-reported survey data may be subject to recall and response bias. Referral increases could have been influenced by external factors, and the one-year follow-up does not capture longer-term outcomes such as screening completion, adherence, or mortality impact. Additionally, the limited number did not allow for subgroup analyses that may identify differences in education efficacy in specific provider and practice types. The surveys administered after participation in the education curriculum session did not include questions assessing overall satisfaction with the curriculum or suggestions for improvement, limiting our ability to capture feedback on curriculum quality or usability. Future studies will incorporate human-centered design methods and engage primary care providers as co-designers to iteratively refine the curriculum and integrate structured feedback from providers across clinics within the safety-net hospital system. Finally, our study did not specifically address patient-centered barriers such as medical mistrust, low perceived cancer risk, and health literacy, which could be accomplished through community partnerships and public health outreach. In addition to provider-focused strategies, direct consumer education is critical to empowering patients to initiate conversations with providers about lung cancer screening. Our team has evaluated a patient outreach strategy using multimodal education approaches outside the clinical encounter, for which findings from this work are forthcoming. Combining provider education with multi-level engagement strategies may offer the most comprehensive path toward improving LCS participation for those at highest risk and reducing lung cancer-related disparities.

## 5 Conclusion

LCS with LDCT reduces mortality by early detection of lung cancer (4). This pilot study demonstrates that a focused, 30-minute hybrid in-person and virtual educational curriculum for primary care providers (PCPs) can effectively improve provider knowledge of LCS eligibility criteria, comfort in SDM conversations, confidence in managing LDCT results, and accuracy in applying screening guidelines to diverse patient scenarios in a safety-net hospital primary care setting. Importantly, the curriculum led to a substantial increase in referrals of screening-eligible patients, reinforcing that PCP education is both feasible and impactful. These findings support the integration of targeted, team-based education as a critical component of broader efforts to increase equitable LCS uptake in real-world primary care settings. Future large, multicenter studies are needed to confirm these findings and further refine an educational curriculum and identify addressable limitations to increase LCS uptake.

## Data Availability

The raw data supporting the conclusions of this article will be made available by the authors, without undue reservation.
